# Diet in Disease

**Published:** 1893-02-11

**Authors:** Ernest Hart

**Affiliations:** Bachelier-ès-Sciences-es-Lettres (rèstreint), formerly Student of the Faculty of Medicine of Paris, and of the London School of Medicine for Women


### Diet in Disease.

By Mrs. Ernest Hart, Bachelier-ès-Sciences-es-Lettres (rêstreint), formerly Student of the Faculty of Medicine of Paris, and of the London School of Medicine for Women.

### XII.—DIABETES.

Having now considered the values of the various foods used by civilised man, and described the processes in the human economy by which these foods are digested, I propose to consider the deflections from the normal in the long and elaborate process of digestion, and the treatment or rectification of these abnormal conditions by diet. In treating of the question of dietetics, I do not propose to simply state the foods which must be avoided, or which may be allowed, but also to arrange the daily *menus* for the patient, and to give practical instructions how the dishes are to be prepared. Diabetes, gout, dyspepsia, &c., their digestive causes and their dietetic treatment, will be treated in order; and I trust to be able to make it plain that a suitable dietary and an intelligent cook are more valuable to patients suffering from these complaints than all the drugs of the pharmacopœia.

**Diabetes** was considered not so long ago to be an incurable and inevitably fatal disease. Thanks, however, to the labours of Claude Bernard, Germain Sée, and Pavy, the cause of this mysterious wasting disease has been discovered to be an inability on the part of the economy to assimilate starch and sugar, and, in the severer cases, in the morbid production of sugar from the tissues themselves. Hence diet and the rigid exclusion of starch and sugar from the food becomes the most important factor in the treatment of diabetes. The value of starch as a food, and its behaviour in the body, has been already described. (October 8th, page 19; December 3rd, page 149; and December 10th, page 165.) The facts are briefly—Starch is indigestible in the uncooked state; cooked it is insoluble, and incapable of passing through the membranes of the blood vessels of the stomach and intestine. To be rendered soluble and capable of assimilation it must be converted into glucose or grape sugar. This is done partly in the mouth, by the action of the diastase of the ptyalin of the saliva, but much more rapidly in the duodenum by the action of the pancreatin of the pancreas. Cane sugar is dissolved by the fluids of the stomach, or is swallowed in a soluble condition. The soluble glucose and sugar are absorbed by the venules of the stomach and intestine, and carried at once by the portal vein to the stomach. In the liver the sugar is lost sight of, and though after the ingestion of food the portal vein may contain an abundance of saccharine matter in solution there is not a trace of sugar in a healthy person in the blood of the hepatic vein. What then becomes of the sugar in the liver? This is a question which has been partially solved for us by the researches of Claude Bernard. He discovered the presence of an amyloid or starchy substance, which he called glycogen, stored in the liver cells, and the result of his researches tends to show that the soluble sugar which is brought to the liver by the portal vein is converted by means of a strange and not well-understood action of the liver cells into an insoluble colloid substance, and is as such lodged and stored up in the cells. It is there available, as Claude Bernard insisted, for conversion again into sugar to be carried off by the capillaries and burnt up in the tissues in the course of tissue change and the production of energy. Claude Bernard contended that the liver was a magazine for the storage of sugar in the form of glycogen, and the regulation of its supply to the economy; without the interposition of the liver, sugar would obviously be introduced into the circulation in irregular quantities at the moment of digestion, which would have a very disturbing influence on the system. It has since, however, been strenuously denied by Pavy that glycogen is ever reconverted into sugar, or that sugar is burnt up in the tissues. Recent researches tend to show that the formation of glycogen in the liver is the first step in the metamorphosis of starch and sugar into fat, and that it is fat and not sugar which is the hydro-carbon burnt up in the tissues. Now it is obvious that if by some morbid change in the liver-cells they have lost the power of arresting the sugar and converting it into glycogen, that the sugar will pass into the general circulation and that it will appear in the urine. This is what takes place in diabetes. The indication is therefore to check the ingestion of starch and sugar. There is also another diabetic indication given by understanding the normal physiological process. If, as is believed, fat is manufactured from glycogen, the emaciation and weakness so characteristic of diabetes are due to the want of fat in the tissues, which fat is not only deposited in a small degree, but is consumed in the production of force. It is therefore obviously necessary that as we deprive the diabetic of the hydrocarbons of starch and sugar in his food we should supply their place with the hydro-carbon of fat.

Diabetes may be divided into (1) glycosuric dyspepsia, (2) diabetes minor, (3) diabetes major.

1. In simple cases of **Glycosuric Dyspepsia** the disorder seems to be functional. It generally rapidly yields to dietetic treatment, and the sugar which may be present in the urine in the first instance in considerable quantities disappears almost completely, if not entirely, by the rigid exclusion of starch and sugar from the diet. In these cases it seems as if the liver had lost its power of converting sugar into glycogen, and that therefore the sugar ingested with the food escaped unchanged into the blood. The excessive thirst, the malaise, and the dyspepsia characteristic of the ma'ady disappear on the enforcement of a rigid diet, but they make their appearance again on any relaxation of the regimen.

2. In **Diabetes Minor** there is probably some permanent impairment of the powers of the liver, and though by the maintenance of a diet devoid of starch and sugar the amount of sugar in the urine may be considerably reduced, it is scarcely ever banished altogether. It is these cases which derive so much benefit from the treatment by alkaline waters, such as is carried out at Carlsbad and Vichy. This form of diabetes is frequently associated with gout, or what is called the uric acid diathesis. The continual maintenance of an exclusive diet has a quite remarkable influence in cases of diabetes minor, and whereas patients who heretofore, before the cause and nature of diabetes were discovered, would drag out a miserable existence, tormented with thirst, growing weaker from increased muscular feebleness and doomed to an early death, can now by a rational dietary, which it is neither painful nor disagreeable to maintain, be kept in fairly good health.

3. **Diabetes Major** generally occurs in young and thin persons, and is a very grave malady. It is little influenced by diet, for though starch and sugar may be excluded and the patient may be kept exclusively on a flesh diet, the liver in this case exercises its power to break up the nitrogenous elements and to extract glycogen from albuminous foods, and even from the tissues of the patient himself, so that the patient is, as it were, devoured by the abnormal activity of his own liver. It is thus seen that similar symptoms are produced by totally opposite conditions. In diabetes minor the glycogenic function of the liver is depressed, and it fails to convert the sugar brought by the portal vein into glycogen. In diabetes major the glycogenic function of the liver is abnormally excited, and the liver cells convert into glycogen even the nitrogenous elements of the muscles of the patient. It is a question whether we have not here to do with two totally different diseases, and whether, owing to the presence of an identical symptom, viz., the presence of sugar in the urine, we do not err in submitting both classes of patients to the same regimen. In the first, the exclusion of starch and sugar is well borne; in the latter, the economy seems to cry out for sugar, in order to feed the rapacious voracity of the liver. The most, however, that can be done in cases of diabetes major is to relieve the sufferings of the patient, and to make the end as easy as possible.

